# Supplementary material for: Content-rich biological network constructed by mining PubMed abstracts
Source: BMC Bioinformatics. 2004 Oct 8;5:147. doi: 10.1186/1471-2105-5-147 (PMC528731; doi:10.1186/1471-2105-5-147)
Supplement: Additional File 5 — The original Chilibot query results of the term "long-term potentiation (LTP)" and 22 other terms, limiting the latest references analyzed to the years 1990, 1995, 2000, and 2004. [file 1471-2105-5-147-S5.bz2 › chilibotAdditionalFile5/ltp2004/html/SYNAPTOTAGMIN_SYNAPTOPHYSIN.html]

 


 **SYNAPTOTAGMIN** and **SYNAPTOPHYSIN** 
  
Found 99 abstracts in PubMed,  **30 abstracts were retrieved and analyzed**.  


---

 Search Google  |
 PDF files only 
|  EDU domain only 

---

**Interactive relationship** (e.g. stimulation, inhibition, etc)

- The results demonstrate that chronic treatment with fluoxetine and tranylcypromine leads to an increased expression of  **synaptophysin** , but decreased expression of  **synaptotagmin**  and VAMP in the hippocampus and cerebral cortex.  Ref: 14708000 Pharmacogenomics JPharmacogenomics J, 2003
- In these investigations we found that the same treatment induced accumulation over 2 fold of other proteins in the secretory pathway, including synaptosomal associated protein of 25 kDa SNAP 25,  **synaptotagmin**  III, synaptobrevin,  **synaptophysin** , and cyclophilin B, and did not affect accumulation of others, including  **synaptotagmin**  I, calnexin, and glucose regulated protein 94.  Ref: 10965922 Endocrinology, 2000

- :-)

  **Parallel relationship** (e.g. studied together, co-existance, homology, etc.)

  - The results demonstrate that stress exposure leads to reduced expression of  **synaptophysin**  but increased expression of  **synaptotagmin**  in the hippocampus.  Ref: 11720700 Biol Psychiatry, 2001
  - There was a down regulation of  **synaptophysin** ,  **synaptotagmin** , and protein phosphatases PP1alpha and PP1beta mRNAs in AD.  Ref: 12462403 Neurochem Res, 2002
  - Furthermore, protein levels of  **synaptophysin** , syntaxin, and  **synaptotagmin**  synaptic proteins, respectively were up regulated by estradiol.  Ref: 12554763 Mol Endocrinol, 2003
  - Many of these factors, which collectively can be called SNARE regulators e.g. Sec1 Munc18,  **synaptotagmin** , GATE 16, LMA1, Munc13 UNC 13,  **synaptophysin** , tomosyn, Vsm1, etc.  Ref: 12914951 Biochim Biophys Acta, 2003
  - Redistribution of alphaS from perikarya to processes of SN neurons occurred by 18 weeks GA in parallel with  **synaptophysin** , while betaS and  **synaptotagmin**  were redistributed similarly between 20 and 28 weeks GA and this also occurred with gammaS and synaptobrevin between 33 weeks GA and 9 months postnatal.  Ref: 11259122 Exp Neurol, 2001
  - Horseradish peroxidase filled vacuoles as wells as vesicles contained the synaptic vesicle membrane proteins VAMP II,  **synaptotagmin** , SV2, and  **synaptophysin** , the vesicle associated proteins rab 3A and synapsin I, and in addition SNAP 25.  Ref: 10579591 Neuroscience, 1999
  - Vesicle factions affinity purified by the CAPS2 antibody from mouse cerebella contained significant amounts of neurotrophin 3 NT 3, brain derived neurotrophic factor BDNF, and chromogranin B but not marker proteins for synaptic vesicle  **synaptophysin**  and  **synaptotagmin** .  Ref: 14715936 J Neurosci, 2004
  - In order to investigate the effects of antidepressant compounds on SVP mRNA levels, the expressions of  **synaptophysin** ,  **synaptotagmin** , VAMP, and synapsin I were analysed by in situ hybridization in rats which had been treated with desipramine, fluoxetine, tranylcypromine, or saline.  Ref: 14708000 Pharmacogenomics JPharmacogenomics J, 2004
  - The amount of immunoreactivity for  **synaptophysin** ,  **synaptotagmin** , and synaptosomal associated protein 25 did not differ between the young and aged rats.  Ref: 10588583 Neurobiol AgingNeurobiol Aging, 2004
  - BDNF also increased the levels of the vesicle proteins  **synaptophysin** , synaptobrevin, and  **synaptotagmin** , without affecting the presynaptic membrane proteins syntaxin and SNAP 25, or the vesicle binding protein synapsin I.  Ref: 11483592 J Biol Chem, 2001
  - N1E 115 cells transfected with necdin cDNA showed morphological differentiation such as neurite outgrowth and expression of the synaptic marker proteins  **synaptotagmin**  and  **synaptophysin** .  Ref: 12198120 J Biol Chem, 2002
  - Proteins in the salivary glands of partially fed female lone star ticks cross react individually with antibodies to synaptobrevin 2 SNARE, syntaxin 1A, syntaxin 2 and SNAP 25 SNAREs, cytosolic alpha beta SNAP and NSF, Ca sensitive  **synaptotagmin** , vesicle associated  **synaptophysin** , and regulatory cell trafficking GTPases Rab3A and nSec1.  Ref: 12429123 Insect Biochem Mol Biol, 2002
  - Components of synaptic vesicles, such as VAMP 2 synaptobrevin II,  **synaptophysin** ,  **synaptotagmin** , or proteins of the perisynaptic plasma membrane such as GABA transporter 1 GAT1, were not present.  Ref: 11182086 Neuron, 2001
  - Interestingly, these changes occurred without apparent changes in synaptic structure, because a number of presynaptic marker mRNAs growth associated protein 43, synapsin,  **synaptophysin** , synaptopodin,  **synaptotagmin** , syntaxin remained stable.  Ref: 12832546 J Neurosci, 2003
  - In addition, scinderin antisense treatment did not alter the expression of proteins involved in vesicle plasma membrane fusion, such as  **synaptophysin** ,  **synaptotagmin**  or syntaxin, indicating a lack of effects on the fusion machinery components.  Ref: 11158248 J Neurochem, 2001
  - By double immunostaining with specific antibodies against  **synaptophysin** ,  **synaptotagmin**  I, VAMP1 and VAMP2, we identified different relative levels of these integral membrane proteins of synaptic vesicles in comparison to boutons of the same rat cortical neuron.  Ref: 12713639 Eur J Neurosci, 2003
  - In parallel, there was a substantial increase in the expression levels of the presynaptic markers  **synaptophysin** ,  **synaptotagmin** , and synapsin I and of GluR1 AMPA receptor subunits.  Ref: 12595243 Mol Cell Neurosci, 2003
  - The current view is that sprouts have the ability to form functional synapses as they display a number of key proteins required for exocytosis SNAP 25, VAMP synaptobrevin, syntaxin I,  **synaptotagmin**  II,  **synaptophysin** , and voltage activated Na, calcium and calcium activated K channels.  Ref: 11755789 J Physiol ParisJ Physiol Paris, 2003
  - Moreover, they were labelled along their entire lengths with antibodies against  **synaptotagmin**  and  **synaptophysin** , but these failed to reveal intraspinal terminal fields.  Ref: 12541316 J Comp Neurol, 2003
  - The amount of immunoreactive synapsin I,  **synaptophysin** , and  **synaptotagmin**  was not appreciably changed for 72 hours after reperfusion.  Ref: 11988617 Stroke, 2002
  - Stress differentially regulates  **synaptophysin**  and  **synaptotagmin**  expression in hippocampus.  Ref: 11720700 Biol Psychiatry, 2001
  - BACKGROUND In view of the effects of stress on synaptic plasticity, the regulation of  **synaptophysin**  and  **synaptotagmin**  expression by immobilization was analyzed by in situ hybridization.  Ref: 11720700 Biol Psychiatry, 2001
  - The delayed increase in  **synaptotagmin**  was blocked by protein synthesis inhibitors, while the early increase in  **synaptophysin**  and synaptobrevin was not.  Ref: 11483592 J Biol Chem, 2001
  - These synucleins first appeared in perikarya of SN neurons after  **synaptophysin** , but about the same time as  **synaptotagmin**  and synaptobrevin.  Ref: 11259122 Exp Neurol, 2001
  - Immunocytochemistry also confirmed the presence of these SNAP receptor SNARE proteins, as well as  **synaptophysin** ,  **synaptotagmin** , and syntaxin.  Ref: 12438157 Ann N Y Acad Sci, 2002
